# Social Media and Health Promotion

**DOI:** 10.3390/ijerph17093323

**Published:** 2020-05-11

**Authors:** Michael Stellefson, Samantha R. Paige, Beth H. Chaney, J. Don Chaney

**Affiliations:** 1 Department of Health Education and Promotion, East Carolina University, Greenville, NC 27858, USA; chaneye@ecu.edu (B.H.C.); chaneyd@ecu.edu (J.D.C.); 2 STEM Translational Communication Center, University of Florida, Gainesville, FL 32611, USA; paigesr190@ufl.edu

With over 3 billion users worldwide, social media has become a staple of daily life for people across the globe. Social media allows virtual network members to quickly cultivate and exchange information and ideas in the form of video, image, text, and multimedia. The success of social media is grounded in its ability to adapt to the dynamic social contexts of its users and evolve with the sophistication of technology. In the health promotion profession, we have recognized the power and success of social media in achieving goals and objectives of public health, including behavioral, organizational, and policy change [1]. However, as health promotion researchers and practitioners, we simply cannot ignore the fact that these powerful tools also present a number of challenges (e.g., managing misinformation) and complications (e.g., ensuring compliance with privacy protections) that may eventually hinder our efforts and become a detriment to public health [2]. Our Special Issue includes a collection of innovative studies to help us better understand these challenges and complications and what they mean for the future of health promotion. 

Unique to other special issues related to this topic is our focus on supplementing traditional approaches of health promotion with principles of translational health communication. We present 11 papers that employ theories of both behavior change and social influence to understand how social media is used by diverse audience segments in various health contexts. Strong attention is placed on the social, physical, and geographic factors that facilitate and hinder its use as an effective behavior change and decision-making tool. Perhaps most notable within the issue is its collective focus on using social media as a dissemination tool and ensuring that current and emerging collaborative technologies are appropriate for the audience(s) and message(s). In our opinion, this Special Issue generates a breadth of new knowledge about social media in health promotion, but, most importantly, it harnesses core principles of two interrelated fields (i.e., health promotion and translational health communication) to demonstrate the depth of the challenges and complications we seek to understand and overcome. In the following paragraphs, we provide a brief synopsis of each article, highlighting its contribution to the aims of our Special Issue.

Kvardova, Machackova, and Smahel [3] used the Tripartite Influence Model, a theoretical framework that explains eating disturbances with socio-cultural factors, to expand knowledge about the role of health-related websites in the development of eating disorders. Among young adult women, the drive for thinness was positively correlated with online social support, web content internalization, as well as neuroticism. These findings confirmed what is hypothesized in the Tripartite Influence Model: body image concerns and eating disorders are directly affected by socio-cultural factors (e.g., media pressures, peer criticism) and indirectly through the internalization of the medialized body ideals. The authors acknowledge the potential impact of health-oriented websites on young women and their drive for thinness, especially in the context of the internalization of body appearance standards; likewise, it is suggested that future research consider how social media influencers can be especially protective or detrimental to women’s perception of body ideals. 

Giorgi and colleagues [4] examined geo-located language in public tweets mentioning the term “drunk” and correlated this language with the prevalence of self-reported excessive alcohol consumption as reported in the United States (U.S.) Behavioral Risk Factor Surveillance System. Linguistic markers associated with excessive drinking were subsequently identified in different regions and cultural communities as identified by the American Community Project. The frequency with which people tweeted the word “drunk” (as a percentage of all tweets) was moderately correlated with excess drinking at both the county and state level. Of particular note, communities differed both in terms of how much they tweet about drinking and how they tweet about drinking. Results showed that tweets about being drunk were predictive of different “styles” of excessive drinking behavior across many types of communities derived from demographic and socio-economic indicators. The particular words, phrases, and linguistic themes associated with alcohol abuse within particular regions and communities can provide insight into sociocultural alcohol use and may help to shape targeted public health messages that recognize the cultural determinants of alcohol use and abuse.

Recognizing that Provincial Health Committees (PHCs) in China have started to adopt the microvideo sharing platform, Tik Tok, to engage with local residents and communicate health-related information, Zhu and colleagues [5] examined 31 verified PHC Tik Tok accounts. Findings suggested that in provinces with greater economic prosperity, local health departments delivered better quality health education to provinces with citizens more likely to have a higher level of health literacy than to provinces with low economic prosperity. The top 100 most liked health communication microvideos were mainly from six PHCs. With the growing number of Tik Tok users, especially among young individuals, the authors suggest that PHCs should continue to refine use of Tik Tok to grow engagement levels with all citizens. Most notably, the authors recommend that use of Tik Tok become part of each PHC’s social media ecosystem that functions to communicate health information to citizens on a more personal level.

Through Natural Language Processing methods, Zhao, Zhang, and Wu [6] investigated five Facebook-based autism support groups. An interactive visualization method (i.e., pyLDAvis) was employed to visualize intertopic distance maps that explored how group members shared information and interacted with one another. In doing so, topics that autism-affected users were most concerned with emerged, along with how these issues were addressed on Facebook. By studying these support groups using text mining and data visualization, researchers were able to gather data on issues that individuals living with autism were concerned about (e.g., parenting, education, and behavior traits). Healthcare professionals can reference this social media data to enhance communication with their patients and informal caregivers. Findings from this study showed that latent Dirichlet allocation is feasible to use when attempting to determine important support topics posted on Facebook autism support groups. 

Using the theories of reasoned action and expectancy confirmation, Wang, Zhang, Zhou, and Lai [7] analyzed the effects of cognitive factors on WeChat users’ health product purchase intentions. In this study, social media services had a higher penetration rate among younger user groups. Trust experienced by customers fully mediated the relationship between emotional price and purchase intention among WeChat users. However, there was no evidence that trust played a mediating role between emotional products and purchase intention. Results indicated that social media has gradually formed an important environment conducive to health communication. However, in order for the public to benefit from such an environment, health service providers must seek to resolve issues related to public mistrust and misinformation on social media.

Trust in online resources also contributes to an individual’s willingness to participate in online support groups found on social media. Through social media content analysis, Apperson and colleagues [8] examined Chronic Obstructive Pulmonary Disease (COPD) self-management information shared within Facebook groups dedicated to the condition. Findings suggested that the purpose of most COPD Facebook groups was to provide support (19/26, 73.1%), while the remaining groups (7/26, 26.9%) built awareness or shared health information. The findings from this study show that members of these Facebook groups shared various experiences managing COPD. Medications, for example, were the most addressed self-management topic on the COPD Facebook groups, while engagement, in the form of “likes”, were highest for posts that demonstrated some form of social support. Overall, the study showed that COPD Facebook group members search for information regarding specific self-management topics and also share their disease-related experiences on the platform. Therefore, use of the social media platform has potential for providing emotional and informational support to users living with COPD. 

Furthering this work, Paige and colleagues [9] drew from social identity theory to examine how communal COPD illness and rural identities influence the degree that a person feels they have online health-related support available to them, should they need it. A survey of social media and clinic-based cohorts demonstrated that socio-demographics, specifically low income and high education, were associated with communal COPD illness identity; however, illness-related experiences (i.e., receiving a physician diagnosis of COPD, identifying as a current/recent smoker) and reporting more severe respiratory symptoms explained the greatest amount of variance in shaping this identity. As expected, a COPD diagnosis and identifying with other patients who live with the condition was associated with a greater degree of available online social support. Interestingly, rural identity moderated the effect of COPD illness identity (and not a COPD diagnosis) on the perceived availability of online social support. This study demonstrates that determining whether social media is the right health promotion tool for a person extends beyond their diagnostic status; rather, there is a need to consider the role of social identities in determining whether social media is an acceptable health promotion and decision-making tool for behavior change. 

With the rapid increase of mobile internet and the emerging popularity of social networks, Gu and colleagues [10] surveyed adults in East China about their use of social media-based health management systems (SocialHMS). These health management systems have been extensively used in patient decision making, chronic disease management, and health information inquiries. One of the great benefits of using SocialHMS is that it provides a convenient method for people to obtain health services. The study explored factors influencing sustained health engagement of SocialHMS while utilizing the theoretical underpinnings of the Theory of Planned Behavior, the big-five theory, and trust theory. Results provided a holistic understanding of the sustained use of SocialHMS both by users and researchers in the context of information systems and healthcare. The authors suggest that social media developers can improve SocialHMS based on individual openness to experience and by matching users to tailored health content based on their respective personality characteristics.

Bopp and colleagues [11] reported on the content, exposure, engagement, and information quality of uploaded physical literacy videos found on YouTube. Over half of the videos demonstrated the concept of physical literacy through unstructured play, otherwise known as “free play”. However, less than half of the videos were deemed to be of high quality according to HONCode guidelines for trustworthy online health information. Videos focusing on physical activity and behaviors had higher overall quality ratings, followed closely by videos addressing affective domains, such as motivation, confidence, and self-esteem. Moreover, the authors assessed the content delivery method and quality. Content and content delivery method were the most significant factors impacting the quality evaluation. Videos that focused on physical activity behaviors had the strongest indication of high-quality ratings, followed by videos covering affective domains of physical literacy. Findings support that YouTube has the potential to enhance video resources; virtual networking opportunities; as well as the sharing, dissemination, accumulation, and enrichment of physical literacy information, especially for youth.

Given the increased use of social media in schools, Bopp and Stellefson [12] provide a critical commentary about challenges and opportunities for using social media to improve physical literacy among youth. Based on the positive relationship between increased physical activity and positive health outcomes, best practices of social media use in the healthcare industry are described for physical educators practicing in schools. Opportunities are discussed for using the ALL-ENGAGE model as a framework for facilitating youth engagement about physical literacy on social media. The authors describe how school administrators should engage with physical educators and the public to address physical activity and misconceptions or misinformation about physical literacy on social media. For example, educators and school systems are encouraged to locate and use social media tools to aid them in enhancing physical literacy among students. Extending upon this recommendation, our commentary, by Stellefson, Paige, Chaney, and Chaney [13], argues that professionals who deliver health education, such as those in public health and school systems, need to be wary of designing and sharing social media interventions or campaigns that are most suited to population segments that are text-, tech- and eHealth-literate. To provide explicit guidance based on our recommendations, we present communication and advocacy roles and responsibilities of health education specialists in the context of social media research and practice. 

The global expansion of social media has resulted in various platforms transforming into promising avenues for the delivery of health promotion messages, self-management education, and interventions. This Special Issue highlights the versatility and flexibility of social media, in that it can be used effectively with a variety of health promotion topics and with many populations (i.e., adolescents, adults, and patients living with a chronic illness). In exploring the depth of challenges and complications related to using social media for health promotion, these studies demonstrate the value of theory- and model-driven approaches in understanding factors that have a fundamental effect on how social media can be used and optimized for health promotion. The factors explored in this Special Issue included socio-cultural identity, trust in online resources, and literacy levels, among others. Of particular note, is that the results of these studies draw our attention to considering a triad of factors associated with health promotion on social media, including what information is exchanged, how it is communicated, and by whom is it is delivered and received. As we consider the potential fit of social media for a particular audience or disease context, we must weigh these factors in addition to the affordances of various online health promotion programs. 

Furthermore, these eleven papers present new opportunities for the development of future social media interventions and analyses. While we believe that researchers and practitioners should tackle these new opportunities head on, it is important to recognize that significant headwinds are likely to come from individuals or entities using social media to promote alternative views on health-related issues or unhealthy behaviors that are not backed by scientific evidence. Therefore, to prevent the spread of health-related misinformation, health education specialists must be vigilant in monitoring and evaluating public health advocacy and communication occurring on social media. The authors in our Special Issue highlight innovative methodologies to efficiently and effectively tackle these endeavors. We firmly believe that results from these studies will expand and build upon traditional health education approaches and improve participative engagement in health promotion through systematic online community building that supports improvements in public health outcomes.
